# LncRNA CHROMR/miR-27b-3p/MET axis promotes the proliferation, invasion, and contributes to rituximab resistance in diffuse large B-cell lymphoma

**DOI:** 10.1016/j.jbc.2024.105762

**Published:** 2024-02-16

**Authors:** Chang Liu, Xinan Zhao, Zifeng Wang, Chan Zhang, Wenbin Zheng, Xiaoxia Zhu, Dong Zhang, Tao Gong, Hong Zhao, Feng Li, Tao Guan, Xiangyang Guo, Hongwei Zhang, Baofeng Yu

**Affiliations:** 1 Department of Biochemistry and Molecular Biology, Changzhi Medical College, Changzhi, Shanxi, China; 2 Department of Biochemistry and Molecular Biology, Shanxi Medical University, Taiyuan, Shanxi, China; 3 Key Laboratory of Cellular Physiology, Shanxi Medical University, Ministry of Education, Taiyuan, China; 4 Cancer Center, Shanxi Bethune Hospital, Shanxi Academy of Medical Sciences, Tongji Shanxi Hospital, Third Hospital of Shanxi Medical University, Taiyuan, China; 5 Central Laboratory, Shanxi Cancer Hospital, Taiyuan, China; 6 Shanxi Hospital Affiliated to Cancer Hospital, Chinese Academy of Medical Sciences, Beijing, China; 7 Cancer Hospital Affiliated to Shanxi Medical University, Taiyuan, China; 8 Department of Hematology, Shanxi Cancer Hospital, Taiyuan, China; 9 Department of Breast Surgery, Shanxi Province Cancer Hospital, Taiyuan, China

**Keywords:** LncRNA CHROMR, miR-27b-3p, MET, rituximab, DLBCL

## Abstract

Long non-coding RNAs (LncRNAs) could regulate chemoresistance through sponging microRNAs (miRNAs) and sequestering RNA binding proteins. However, the mechanism of lncRNAs in rituximab resistance in diffuse large B-cell lymphoma (DLBCL) is largely unknown. Here, we investigated the functions and molecular mechanisms of lncRNA CHROMR in DLBCL tumorigenesis and chemoresistance. LncRNA CHROMR is highly expressed in DLBCL tissues and cells. We examined the oncogenic functions of lncRNA CHROMR in DLBCL by a panel of gain-or-loss-of-function assays and *in vitro* experiments. LncRNA CHROMR suppression promotes CD20 transcription in DLBCL cells and inhibits rituximab resistance. RNA immunoprecipitation, RNA pull-down, and dual luciferase reporter assay reveal that lncRNA CHROMR sponges with miR-27b-3p to regulate mesenchymal-epithelial transition factor (MET) levels and Akt signaling in DLBCL cells. Targeting the lncRNA CHROMR/miR-27b-3p/MET axis reduces DLBCL tumorigenesis. Altogether, these findings provide a new regulatory model, lncRNA CHROMR/miR-27b-3p/MET, which can serve as a potential therapeutic target for DLBCL.

Diffuse large B-cell lymphoma (DLBCL) is the most common subtype of non-Hodgkin’s lymphoma (NHL) with great heterogeneity, accounting for 30-58% of all lymphomas worldwide (1, 2). The standard treatment for DLBCL is the R-CHOP chemotherapy (rituximab plus cyclophosphamide, hydroxydaunorubicin, oncovin and prednisone) (3). Rituximab, the CD20-specific monoclonal antibody, has been approved to treat B-cell malignancies and B-cell-related diseases (4, 5, 6). However, 30-40% of patients have innate resistance to R-CHOP therapy and have eventually become relapsed or refractory (7).

LncRNAs could regulate chemotherapy drug resistance through sponging miRNAs and sequestering RNA binding proteins (8, 9, 10). For example, lncRNA XLOC013218 overexpression promoted temozolomide (TMZ) resistance in glioblastoma multiforme (GBM) by recruiting Sp1 and PIK3R2 (11). LncRNA MALAT1 improved DLBCL chemotherapy sensitivity by upregulating autophagy-related proteins (12). An apoptotic protease-activating factor 1 (APAF1)-binding lncRNA markedly sensitized gastric cancer cells to chemotherapy (13).

Here, we focused on lncRNA CHROMR (cholesterol homeostasis regulator of miRNA expression, ENSG00000223960), also called CHROME or PRKRA-AS1, which is located at 2q31.2 and has five exons. LncRNA CHROMR was overexpressed in rituximab-resistant DLBCL cell lines with the logFC (resistant-sensitive) of 3.43 by bioinformatics analysis in the GSE159852 dataset (7). However, the biological significance and molecular mechanism of lncRNA CHROMR have not been further revealed and thus interested us. LncRNA CHROMR was a crucial mediator in suppressing viral infection of macrophages by reducing interferon regulatory factor (IRF)-dependent signaling (14). LncRNA CHROMR was necroptosis-related with the prognosis prediction of stomach adenocarcinoma (STAD) patients (15), and was m^6^A-related with the prognosis prediction of chronic myeloid leukemia (CML) patients and acute myeloid leukemia (AML) patients by bioinformatics analysis (16). Furthermore, lncRNA CHROMR was able to regulate the efflux of cholesterol and hepatic HDL (high-density lipoproteins) biogenesis by inhibiting miRNAs (17, 18). To elucidate the role of lncRNAs in rituximab-resistant DLBCL cells, it is essential to seek new approaches to overcome rituximab resistance in DLBCL.

In this study, we found that lncRNA CHROMR was expressed at high levels in rituximab-resistant DLBCL cells and DLBCL tissues. LncRNA CHROMR suppression ceased proliferation and invasion of DLBCL cells and promoted cell apoptosis and rituximab resistance. LncRNA CHROMR targets MET by sponging with miR-27b-3p. Our data increases the exploration of lncRNA CHROMR to overcome rituximab resistance in DLBCL.

## Results

### LncRNA CHROMR is highly expressed in rituximab-resistant DLBCL cells and DLBCL tissues

**Figure 1:**
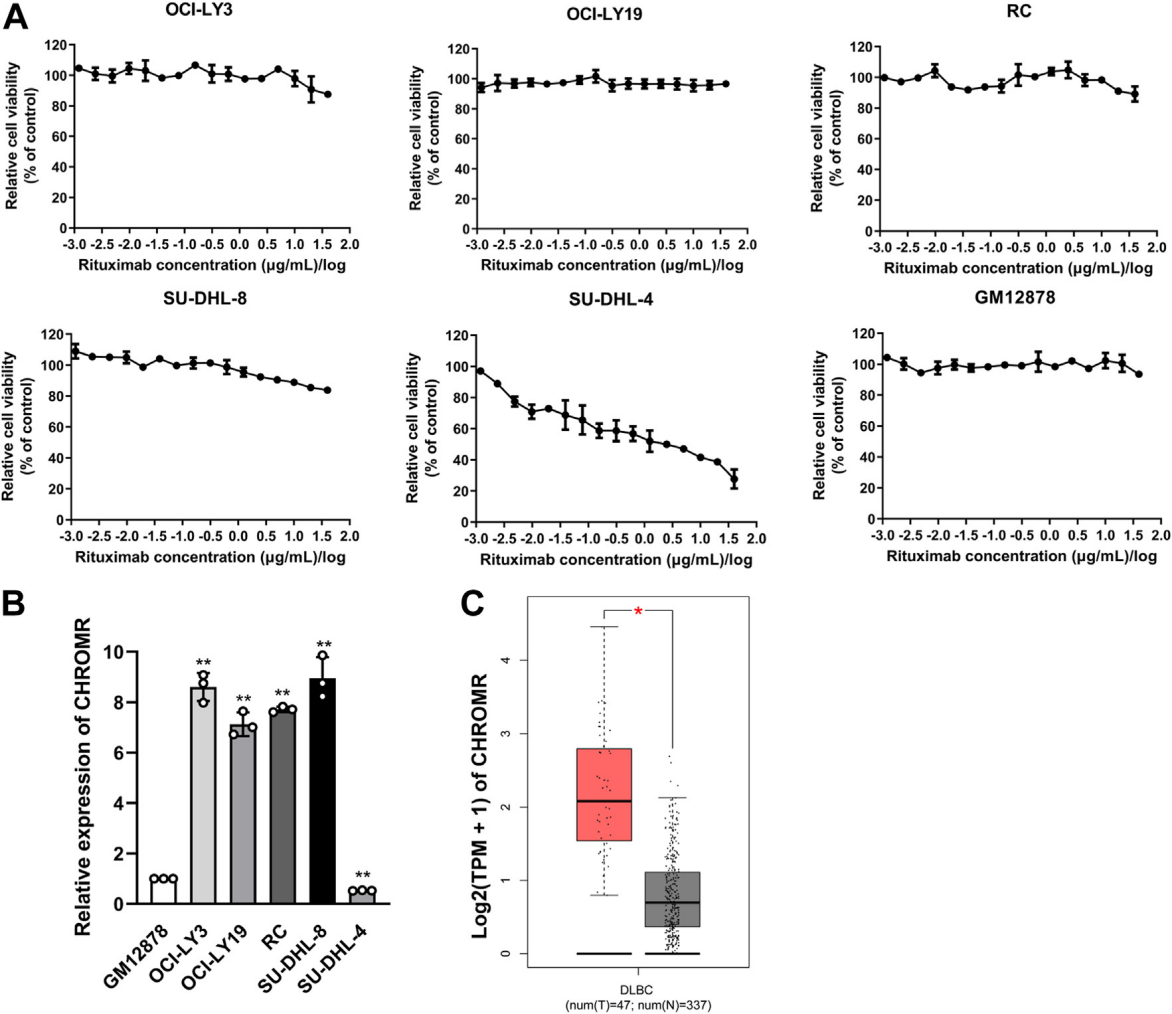
**LncRNA CHROMR is highly expressed in rituximab-resistant DLBCL cells and DLBCL tissues.***A*, the efficacy of rituximab against different DLBCL cell lines and human B lymphocytes GM12878 by CCK-8 assay. *B*, LncRNA CHROMR expression in DLBCL cell lines and human B lymphocytes GM12878 were measured by RT-qPCR. GAPDH was used as endogenous control. Data were demonstrated as mean ± standard deviation of three independent experiments. *C*, expression of lncRNA CHROMR in 47 DLBCL tissues and 337 corresponding para-tumor specimens by TCGA. ∗*p* < 0.05; ∗∗*p* < 0.01.

The dose–response curves of DLBCL cells (OCI-LY3, OCI-LY19, RC, SU-DHL-8, and SU-DHL-4) and human B lymphocytes (GM12878) treated with indicated concentrations of rituximab were depicted in Figure 1*A* by CCK-8 assay. GM12878, OCI-LY3, OCI-LY19, RC, and SU-DHL-8 cells were resistant to rituximab, while SU-DHL-4 was sensitive to rituximab with IC_50_ < 1 μM. LncRNA CHROMR was highly expressed in rituximab-resistant DLBCL cells (OCI-LY3, OCI-LY19, RC, and SU-DHL-8) compared with human B lymphocytes (Fig. 1*B*). LncRNA CHROMR was highly expressed in 47 DLBCL tissues compared to 337 corresponding para-tumor specimens (Fig. 1*C*) by analysis of the TCGA data. Elevated lncRNA CHROMR expression was not correlated with poor survival in DLBCL patients (*p* = 0.69, Fig. S1). However, in the first 50 months, significant correlation can be observed (Fig. S1).

### LncRNA CHROMR suppression enhances CD20 expression

**Figure 2:**
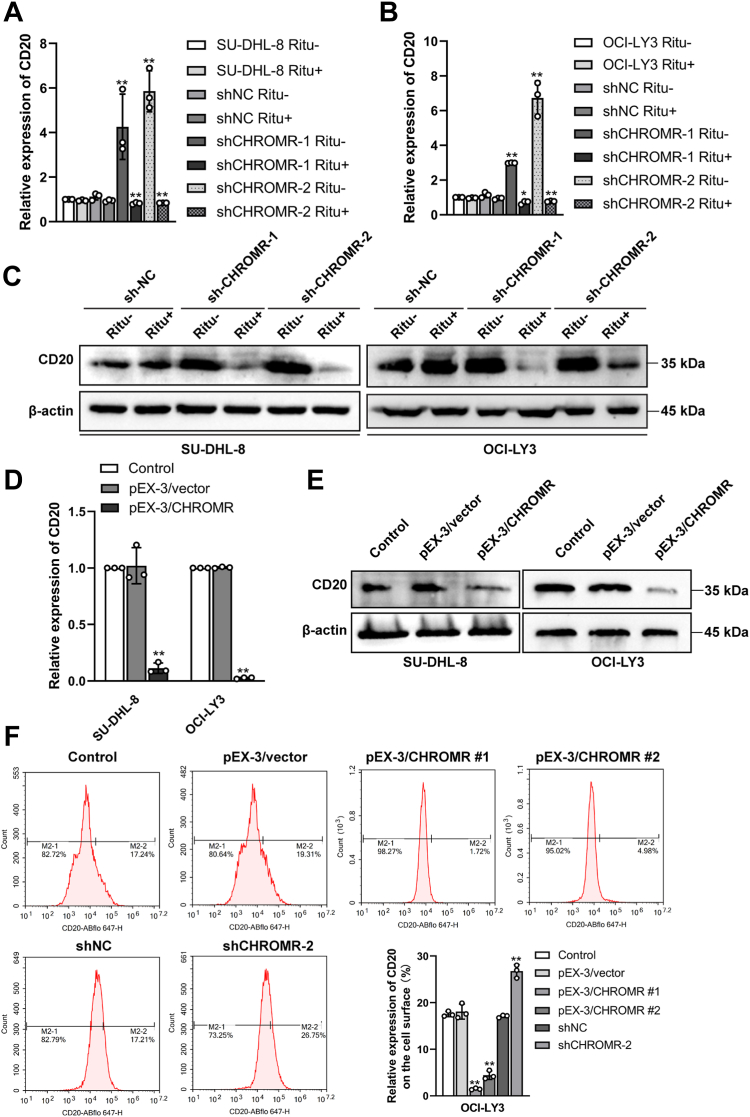
**LncRNA CHROMR suppression elevates CD20 expression in DLBCL cells.** CD20 expression was significantly elevated in the shCHROMR group without the treatment of rituximab and was marked reduced after rituximab treatment by (*A* and *B*) RT-qPCR and (*C*) western blotting. LncRNA CHROMR overexpression inhibited CD20 expression by (*D*) RT-qPCR and (*E*) western blotting. *F*, CD20 expression on the cell surfaces in the indicated groups was examined by flow cytometry. IgG isotype was set as the control. Mean ± SD for three independent experiments are demonstrated. ∗*p* < 0.05; ∗∗*p* < 0.01.

Rituximab targeted CD20-expressing B-cells by indirect or direct binding mechanisms (19). CD20 expression was significantly elevated in the shCHROMR group compared with the shNC group without the treatment of rituximab, and was marked reduced in the shCHROMR group after the treatment of rituximab (20 μg/ml) by RT-qPCR (Fig. 2, *A* and *B*) and western blotting (Fig. 2*C*), suggesting that lncRNA CHROMR suppression enhanced CD20 expression and sensitized DLBCL cells to rituximab. No significant changes in the expression levels of CD20 were observed with or without rituximab treatment in the shNC groups (Fig. 2, *A*–*C*). Meanwhile, lncRNA CHROMR overexpression reduced CD20 expression in SU-DHL-8 and OCI-LY3 cells by RT-qPCR (Fig. 2*D*) and Western blotting (Fig. 2*E*). To evaluate whether lncRNA CHROMR affected CD20 expression on the cell surface, we collected indicated cells and stained. IgG isotype was set as the control. LncRNA CHROMR overexpression or suppression significantly reduced or elevated CD20 expression (Fig. 2*F*). Collectively, lncRNA CHROMR modulated the transcription of CD20 in DLBCL cells.

### LncRNA CHROMR might regulate CD20 expression by phosphorylating HDAC3 in DLBCL cells

**Figure 3:**
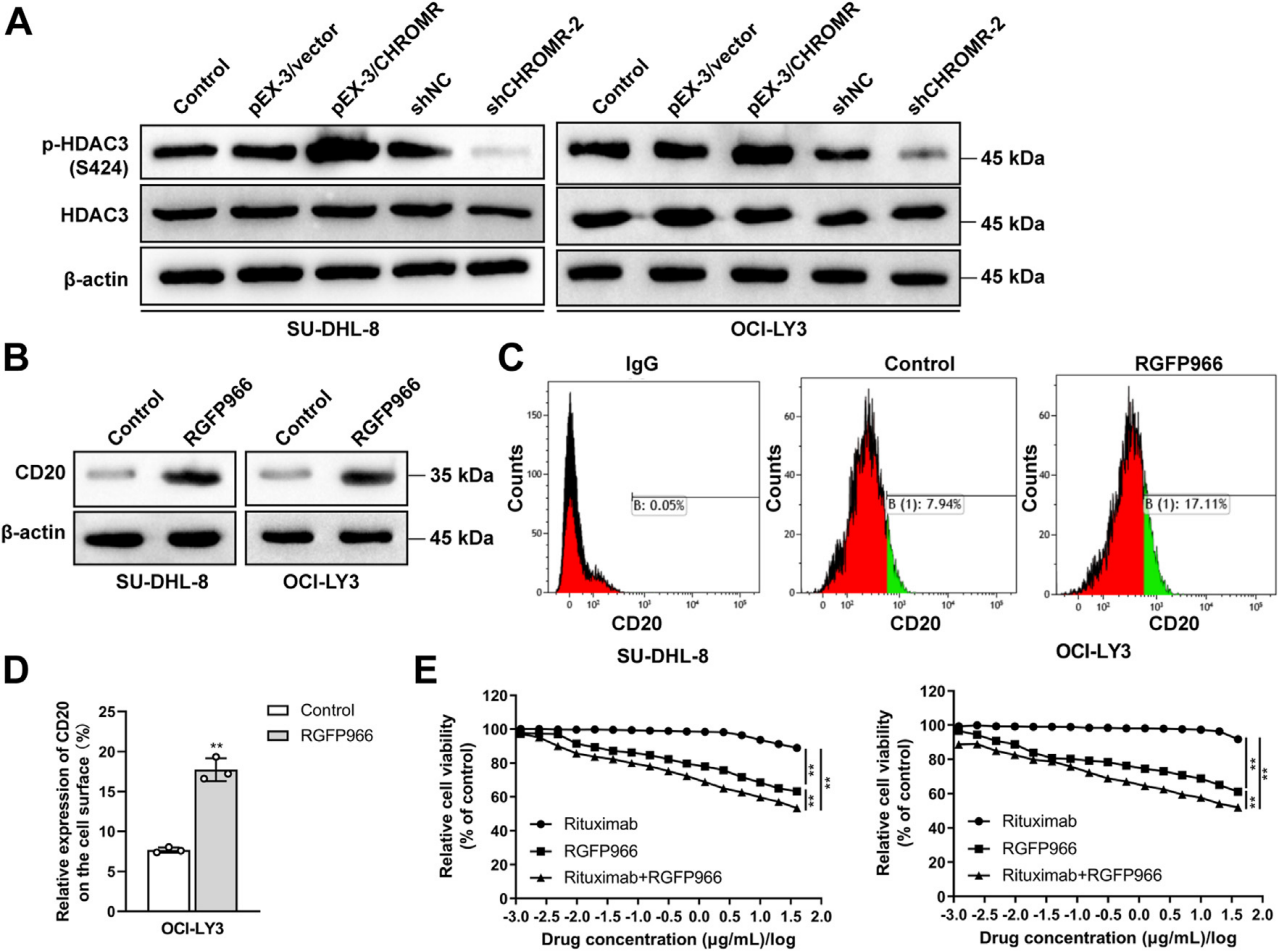
**LncRNA CHROMR might regulate CD20 expression by phosphorylating HDAC3 in DLBCL cells.***A*, the phosphorylation status of HDAC3 in lncRNA CHROMR-overexpressed DLBCL cells and lncRNA CHROMR-depleted DLBCL cells by western blotting. The expression levels of CD20 after the treatment of RGFP966 in DLBCL cells were examined by western blotting (*B*). *C* and *D*, CD20 expression on the cell surfaces in the indicated groups were examined by flow cytometry. IgG isotype was set as the control. *E*, dose–response curves of DLBCL cells to the treatment of RGFP966 alone, rituximab alone, or RGFP966 in combination with rituximab for 72 h. Mean ± SD for three independent experiments are demonstrated. ∗*p* < 0.05; ∗∗*p* < 0.01.

We further examined how lncRNA CHROMR regulates CD20 expression. Previous studies demonstrated that HDAC inhibitors can upregulate the expression of CD20 (20, 21). Then, we wonder whether there is a correlation between lncRNA CHROMR and HDAC. Based on the RPISeq database (http://pridb.gdcb.iastate.edu/RPISeq/), the interaction probabilities between lncRNA CHROMR and HDAC3 were listed as follows: RF (Random Forest) = 0.7, SVM (Support Vector Machine) = 0.97, suggesting lncRNA CHROMR transcript had a high affinity with HDAC3 since RF > 0.5 and 0.57 < SVM < 0.99. The phosphorylation levels of HDAC3 were enhanced upon lncRNA CHROMR overexpression and reduced upon lncRNA CHROMR suppression by western blotting (Fig. 3*A*), while the expression levels of HDAC3 remained almost unchanged. The above results suggested that lncRNA CHROMR might phosphorylate HDAC3. Then, we wondered whether HDAC3 regulated by lncRNA CHROMR further modulated CD20 expression in DLBCL cells. HDAC3 inhibitor RGFP966 (3.6 μg/ml, 10 μM) was used to treat SU-DHL-8 and OCI-LY3 cells for 24 h, and the expression levels of CD20 were significantly elevated after the treatment of RGFP966 by western blotting (Fig. 3*B*). To evaluate whether RGFP966 affected CD20 expression on the cell surface, we collected indicated cells and stained. RGFP966 significantly elevated CD20 expression on the DLBCL cell surface (Fig. 3, *C* and *D*). Further, we treated DLBCL cells with indicated concentrations of RGFP966 alone, rituximab alone, or RGFP966 in combination with rituximab for 72 h to examine whether RGFP966 sensitized DLBCL cells to the treatment of rituximab. The two drug combination was more potent than the single drug treatment in SU-DHL-8 and OCI-LY3 cells (Fig. 3*E*). Collectively, lncRNA CHROMR might regulate CD20 expression by phosphorylating HDAC3 in DLBCL cells.

### LncRNA CHROMR suppression reverses rituximab resistance and inhibits the invasive abilities of DLBCL cells

**Figure 4:**
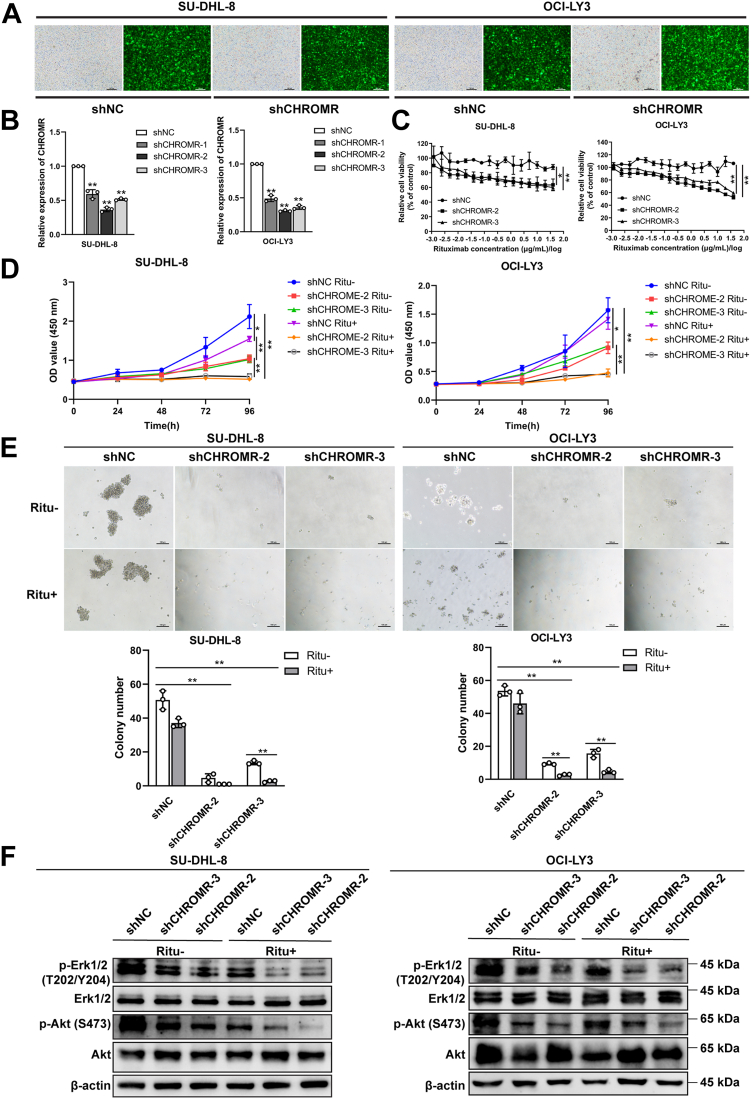
**LncRNA CHROMR is an oncogenic lncRNA in DLBCL cells.***A*, the *green fluorescence* intensity of DLBCL cells transfected with shCHROMR or shNC. *B*, LncRNA CHROMR suppression in SU-DHL-8 and OCI-LY3 was confirmed by RT-qPCR. *C*, the DLBCL cell proliferative ability was evaluated in the presence of the indicated concentrations of rituximab by CCK-8. The DLBCL cell proliferative ability was examined by (*D*) CCK-8 and (*E*) colony formation assay. *F*, the phosphorylation status of signaling proteins in DLBCL cells transfected as A were detected by western blotting. Scale bar: 100 μm. Mean ± SD for three independent experiments are demonstrated. ∗*p* < 0.05; ∗∗*p* < 0.01.

**Figure 5:**
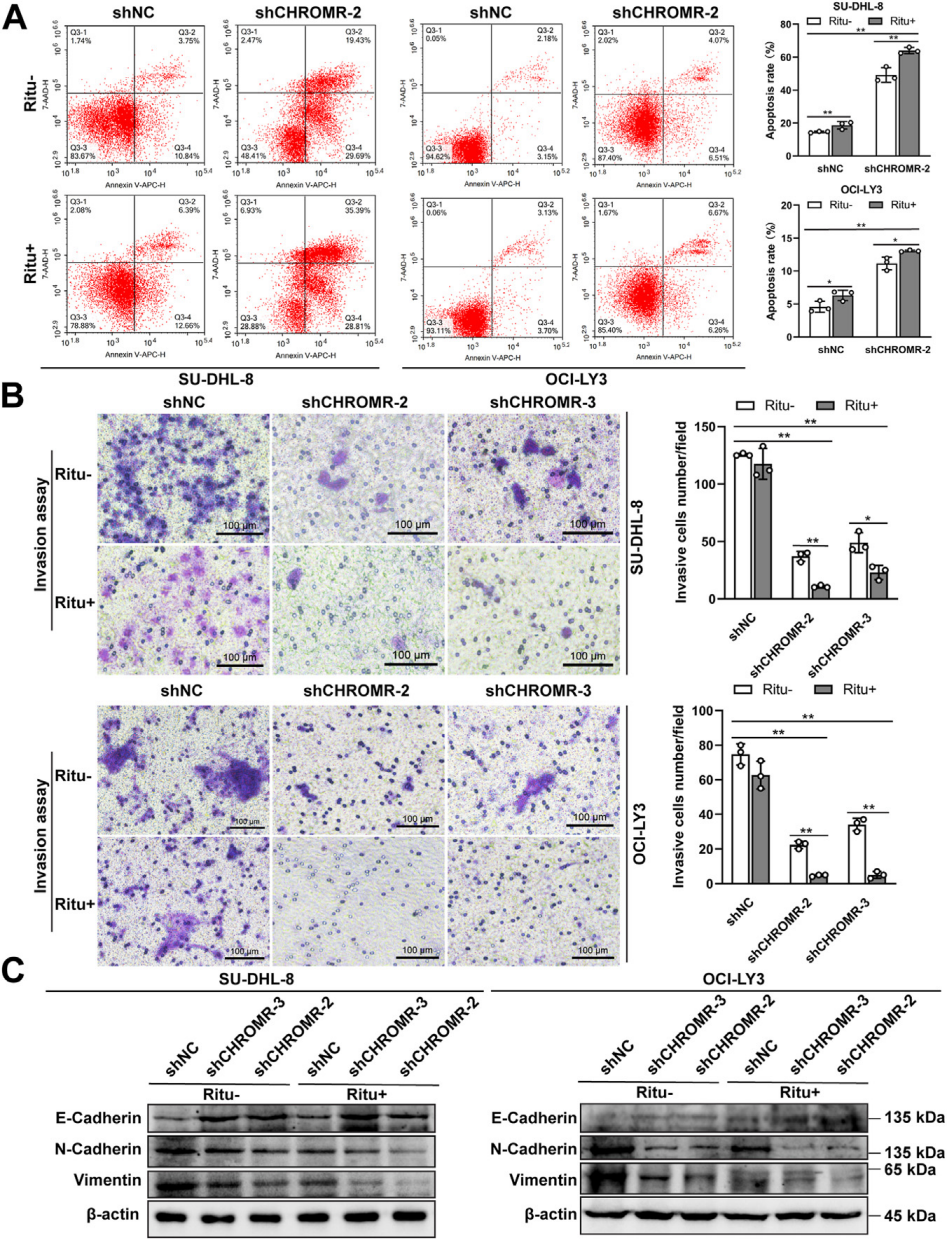
**The roles of lncRNA CHROMR suppression in apoptosis and invasion of DLBCL cells.***A*, cell apoptosis of DLBCL cells transfected with shCHROMR or shNC was measured by Annexin V-APC and 7-AAD staining, followed by flow cytometry. *B*, invasion ability of DLBCL cells transfected with shCHROMR or shNC was measured by transwell invasion assay. Scale bar: 100 μm. Mean ± SD for three independent experiments are demonstrated. ∗*p* < 0.05; ∗∗*p* < 0.01. *C*, the expression of EMT marker proteins in SU-DHL-8 and OCI-LY3 cells transfected as B were detected by western blotting.

We wondered whether lncRNA CHROMR suppression could reverse the resistance of DLBCL cell lines to rituximab. Two stable DLBCL cell lines (SU-DHL-8 and OCI-LY3) using shRNA vectors to regulate lncRNA CHROMR suppression were constructed, verified by the green fluorescence intensity (above 80%) (Fig. 4*A*) and RT-qPCR results (Fig. 4*B*). LncRNA CHROMR suppression reduced the cell growth and rituximab resistance by CCK-8 assay (Fig. 4, *C* and *D*) and colony formation assay (Fig. 4*E*). The activation of MAPK and PI3K/Akt/mTOR signaling pathways is closely correlated with B cell survival (22). The phosphorylation levels of Erk1/2 and Akt were reduced upon lncRNA CHROMR suppression and further downregulated after rituximab treatment (20 μg/ml) (Figs. 4*F* and S2). Moreover, cell apoptosis was promoted upon lncRNA CHROMR suppression, and further elevated by rituximab treatment (20 μg/ml) (Fig. 5*A*). Cell invasive abilities were reduced upon lncRNA CHROMR suppression and further inhibited by rituximab treatment (20 μg/ml) (Fig. 5*B*), evidenced by the downregulation of mesenchymal markers (N-cadherin and vimentin) and upregulation of E-cadherin (Figs. 5*C* and S3). Rituximab treatment (20 μg/ml) further reduced the expression of N-cadherin and vimentin, whereas promoted the expression of E-cadherin in SU-DHL-8 and OCI-LY3 cells (Figs. 5*C* and S3). Collectively, our results show that lncRNA CHROMR is necessary for DLBCL cell proliferation, apoptosis, invasion and rituximab resistance *in vitro*.

### miR-27b-3p is a direct target of lncRNA CHROMR

**Figure 6:**
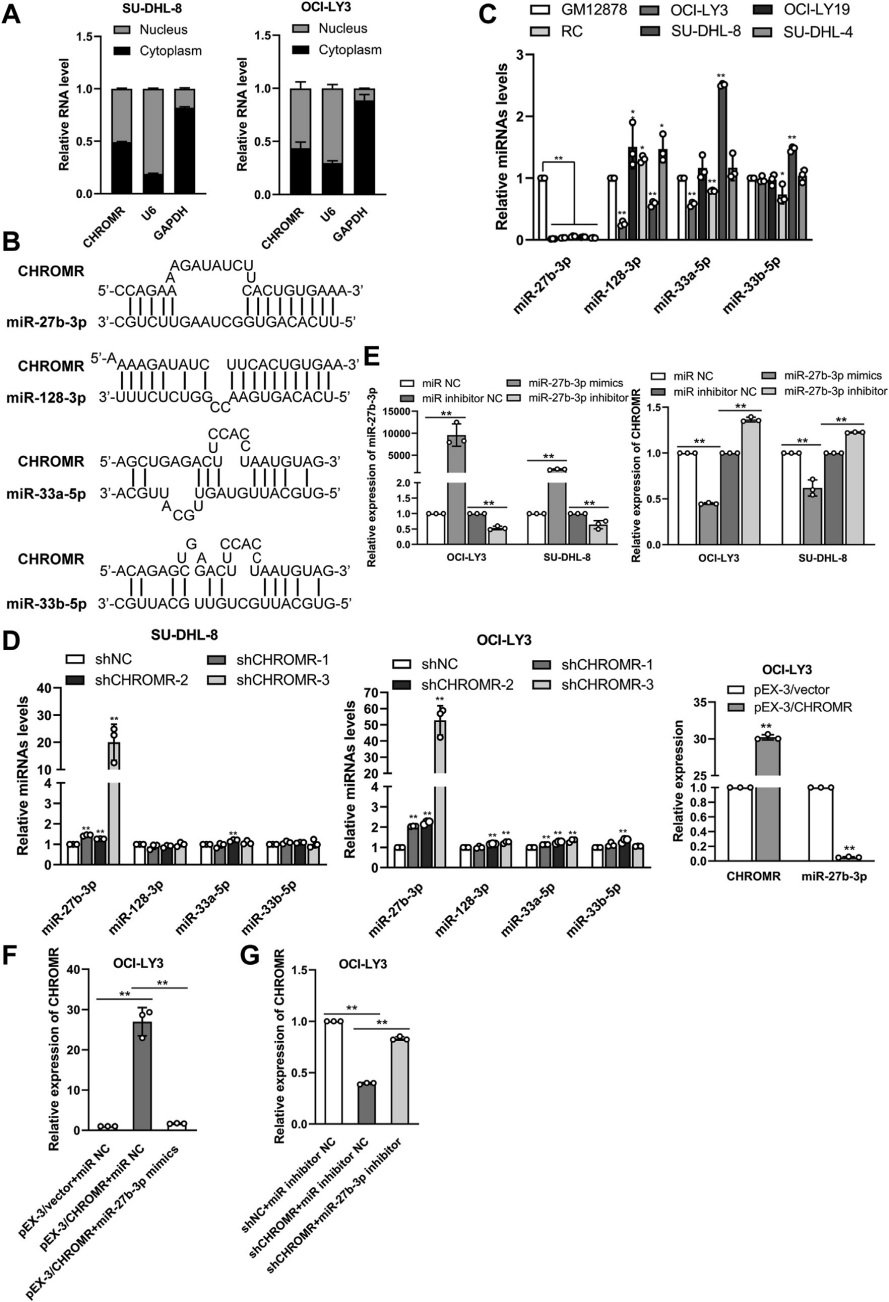
**Interaction between lncRNA CHROMR and miR-27b-3p in DLBCL.***A*, RT-qPCR assay of the nuclear and cytoplasmic distribution of lncRNA CHROMR in DLBCL cell lines. *B*, the top miRNAs interacting with lncRNA CHROMR were predicted by bioinformatics analysis. *C*, MiR-27b-3p, miR-128-3p, miR-33a-5p, and miR-33b-5p expressions in DLBCL cells were examined by RT-qPCR. *D*, effect of lncRNA CHROMR suppression on miR-27b-3p, miR-128-3p, miR-33a-5p, and miR-33b-5p levels in DLBCL cells and effect of lncRNA CHROMR overexpression on miR-27b-3p. *E*, alteration of lncRNA CHROMR levels in DLBCL cells after miR-27b-3p mimics or inhibitor transfection. *F* and *G*, the lncRNA CHROMR levels were determined by RT-qPCR after OCI-LY3 cells were transfected with indicated mimics/inhibitors, shRNAs, or plasmids. Mean ± SD for three independent experiments are demonstrated. ∗*p* < 0.05; ∗∗*p* < 0.01.

**Figure 7:**
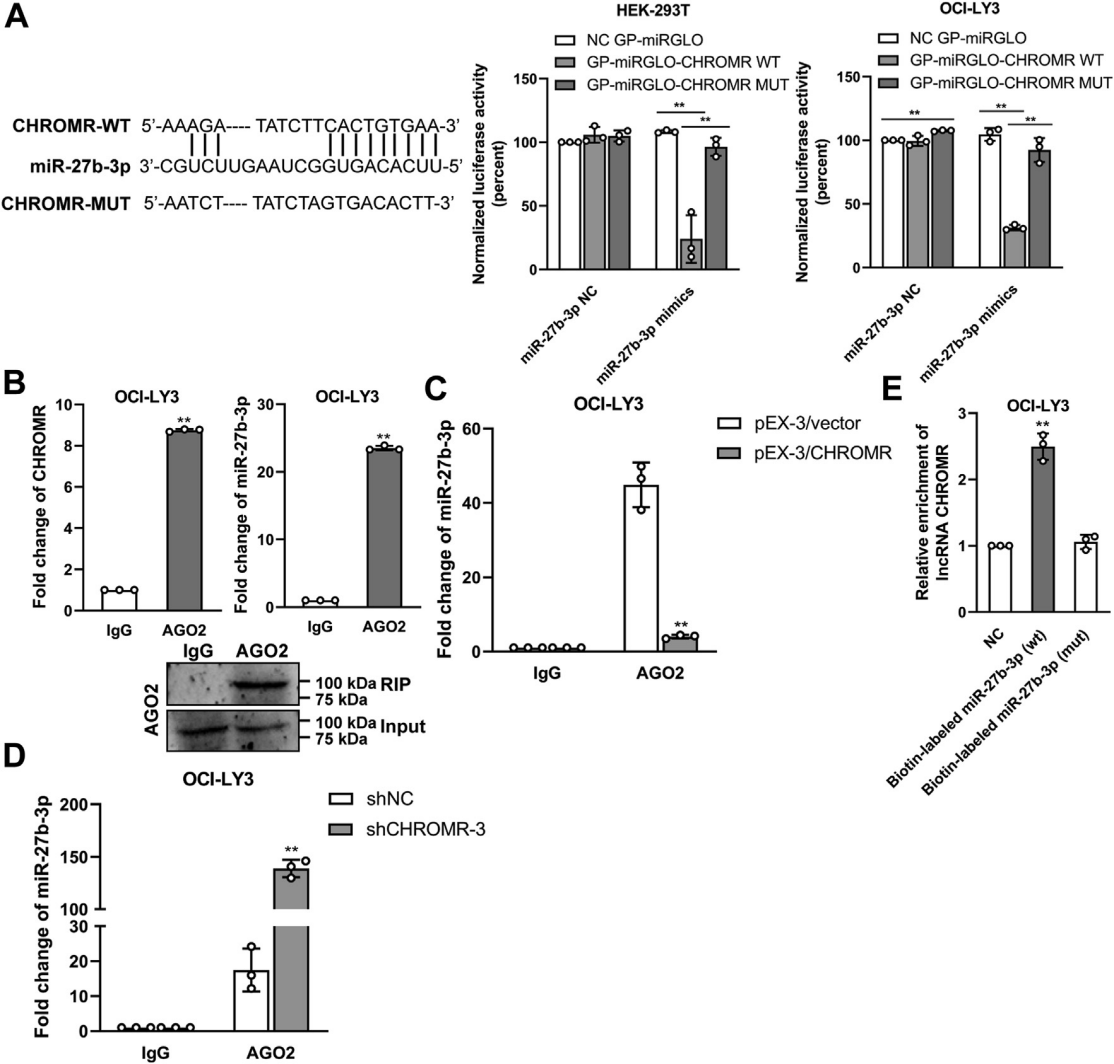
**LncRNA CHROMR sponges with miR-27b-3p in DLBCL.***A*, Luciferase activity in DLBCL cells following the indicated transfection. RIP assay was performed in (*B*) OCI-LY3 cells, (*C*) lncRNA CHROMR-overexpressed OCI-LY3 cells, (*D*) lncRNA CHROMR-depleted OCI-LY3 cells, followed by RT-qPCR to determine bound lncRNA CHROMR and miR-27b-3p associated with AGO2. *E*, RNA pull-down assay followed by biotin-labeled miR-27b-3p (wt or mutant) to examine whether lncRNA CHROMR endogenously associated with miR-27b-3p. Mean ± SD for three independent experiments are demonstrated. ∗*p* < 0.05; ∗∗*p* < 0.01.

LncRNA CHROMR was located both in the cytoplasm and the nucleus (Fig. 6*A*), suggesting that lncRNA CHROMR may exert its molecular function *via* both cytoplasmic and nuclear pathways. Considering its cytoplasmic and nuclear localization, we proposed lncRNA CHROMR may function *via* the ceRNA mechanism. To examine this hypothesis, we predicted the potential miRNAs targeted by lncRNA CHROMR. MiR-33a-5p, miR-33b-5p, miR-27b-3p, and miR-128-3p were the top miRNAs interacting with lncRNA CHROMR by miRTarget2 algorithm (17) (Fig. 6*B*). To verify the bioinformatics prediction, the expression of these four miRNAs in DLBCL cells was determined, and miR-27b-3p expression was significantly reduced in DLBCL cells (Fig. 6*C*). Then, we hypothesized that a correlation may exist between lncRNA CHROMR and miR-27b-3p. Suppression or overexpression of lncRNA CHROMR significantly elevated or reduced miR-27b-3p expression in SU-DHL-8 and OCI-LY3 cells (Fig. 6*D*). Moreover, overexpression or suppression of miR-27b-3p markedly reduced or upregulated lncRNA CHROMR expression (Fig. 6*E*). Finally, lncRNA CHROMR expression was rescued in DLBCL cells after co-transfected with lncRNA CHROMR inserts and miR-27b-3p mimics (Fig. 6*F*) or co-transfected with shCHROMR and miR-27b-3p inhibitor (Fig. 6*G*). MiR-27b-3p reduced the luciferase activity of the lncRNA CHROMR-WT (wild type), but not the lncRNA CHROMR-MUT (mutant) or NC, verifying that miR-27b-3p interacted with lncRNA CHROMR at the predicted binding site (Fig. 7*A*). Based on these results, we examined whether lncRNA CHROMR targets miR-27b-3p to suppress RISC (RNA-induced silencing complex)-mediated downstream mRNA silencing. RIP and RNA pull-down assays were applied to examine this hypothesis. Ago2 binds mature miRNAs to create the RISC (23), and then the RIP assay was conducted to examine whether lncRNA CHROMR and miR-27b-3p are in the same RISC. Both lncRNA CHROMR and miR-27b-3p were highly enriched in the anti-Ago2 group than in the IgG group (Fig. 7*B*). LncRNA CHROMR reduced the binding of miR-27b-3p to AGO2, evidenced by the level of miR-27b-3p was lower in the anti-Ago2 group of the lncRNA CHROMR-overexpressed OCI-LY3 cells than that in the control cells (Fig. 7*C*) and higher in the anti-Ago2 group of the lncRNA CHROMR-depleted OCI-LY3 cells than that in the control cells (Fig. 7*D*). Moreover, biotin-labeled miR-27b-3p was conducted to pull down lncRNA CHROMR, and higher enrichment of lncRNA CHROMR was observed in the complexes sedimented by the wild-type miR-27b-3p, but not by the mutant miR-27b-3p (Fig. 7*E*). The data revealed that lncRNA CHROMR sponges with miR-27b-3p in DLBCL.

**Figure 8:**
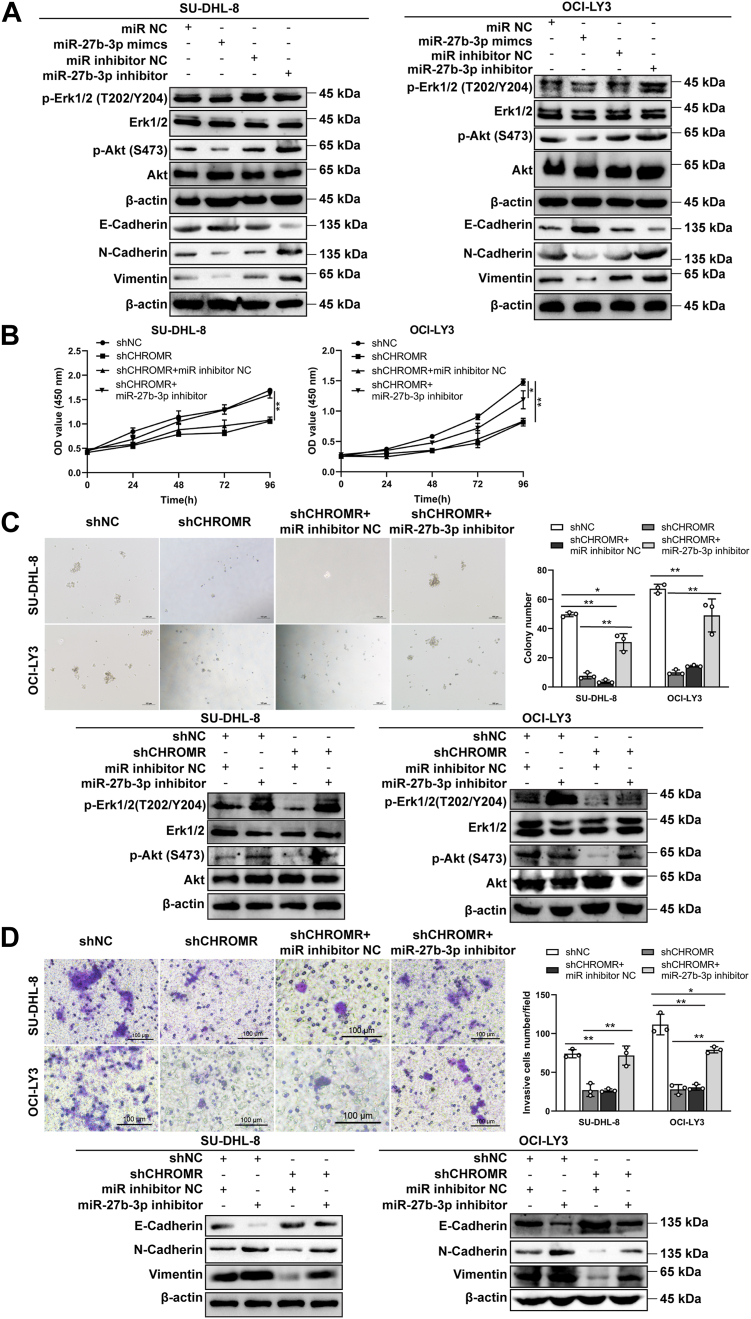
**The roles of lncRNA CHROMR in tumorigenesis of DLBCL cells are dependent on the inhibition of miR-27b-3p.***A*, the phosphorylation status of signaling proteins and the expression of EMT marker proteins in DLBCL cells transfected with miR-27b-3p mimics or inhibitors were detected by western blotting assay. β-actin was used as the internal control for total proteins. DLBCL cells were transfected with indicated shRNAs or inhibitors. The cell proliferative ability was examined by (*B*) CCK-8 and (*C*) colony formation assays. The phosphorylation status of signaling proteins in DLBCL cells was detected by western blotting. *D*, the cell invasive ability was examined by transwell invasion assay. Scale bar: 100 μm. The expression of EMT marker proteins in DLBCL cells was detected by western blotting. Mean ± SD for three independent experiments are demonstrated. ∗*p* < 0.05; ∗∗*p* < 0.01.

miR-27b-3p, a tumor suppressor in DLBCL, was predicted here as a candidate target of lncRNA CHROMR. Then, we examined the effect of miR-27b-3p on the phosphorylation levels of the downstream proteins and EMT-related proteins in SU-DHL-8 and OCI-LY3 cells. MiR-27b-3p overexpression reduced both the phosphorylation levels of Akt and Erk (Figs. 8*A* and S4), the expression of mesenchymal markers (N-cadherin and vimentin), whereas elevated the expression of epithelial marker E-cadherin (Fig. 8*A*). LncRNA CHROMR suppression reduced cell proliferative (Fig. 8, *B* and *C*) and invasive (Fig. 8*D*) abilities, whereas miR-27b-3p inhibitor significantly rescued these phenotypes (Fig. 8, *B*–*D*). Consistently, the phosphorylation levels of Erk1/2 and Akt, and the expression levels of N-cadherin, vimentin, and E-cadherin were rescued in DLBCL cells after co-transfected with shCHROMR and miR-27b-3p inhibitor (Fig. 8, *B*–*D*). Collectively, miR-27b-3p abolishes the roles of lncRNA CHROMR in the proliferation and invasion of DLBCL cells.

### LncRNA CHROMR targets MET by sponging with miR-27b-3p

**Figure 9:**
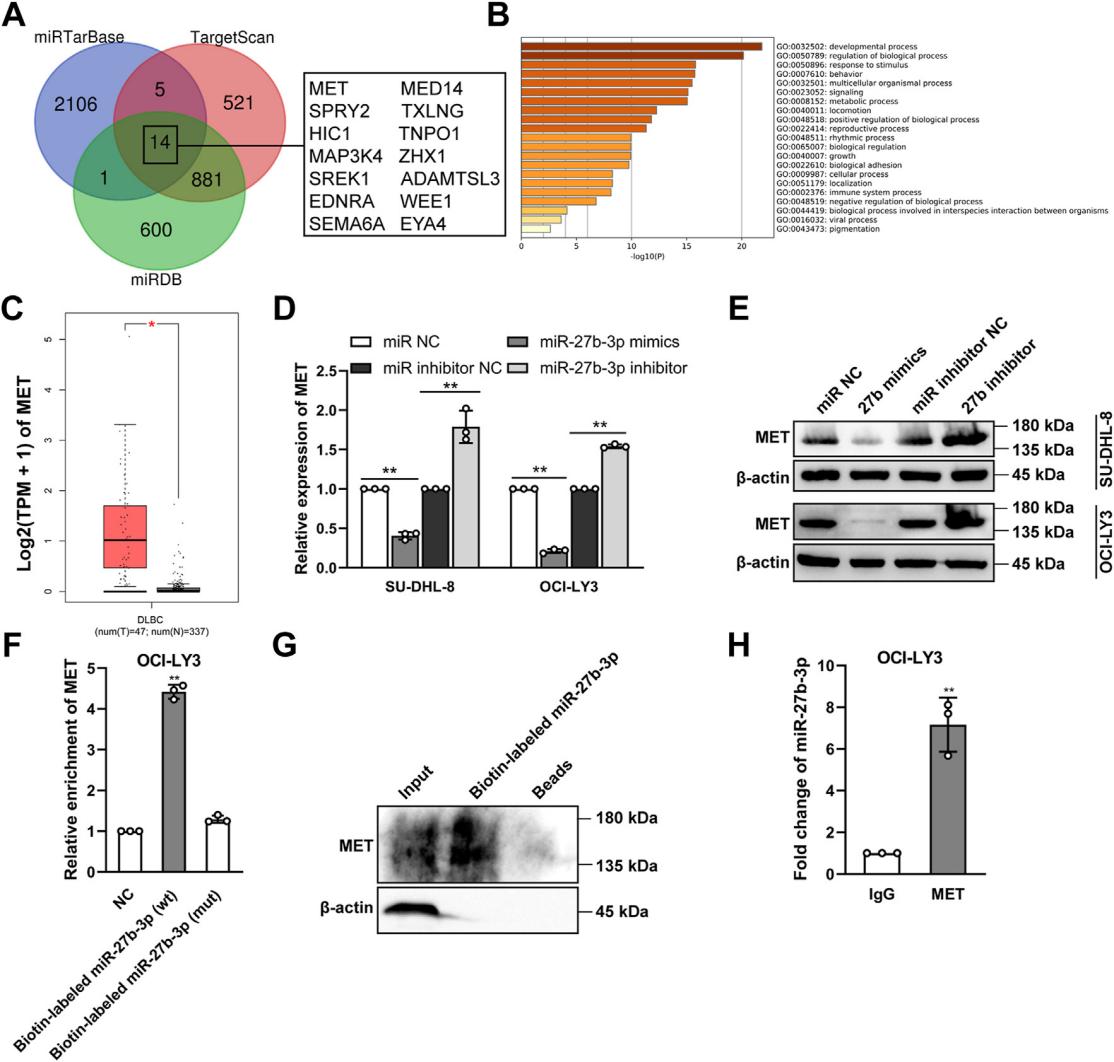
**MET might be a direct target of miR-27b-3p.***A*, target genes for miR-27b-3p were predicted by miRDB, miRTarBase, and TargetScan. *B*, the targeted gene function and pathways of miR-27b-3p were enriched through Metascape. *C*, the expression of MET was analyzed by TCGA data. *D* and *E*, effect of miR-27b-3p on MET levels in DLBCL cells. *F* and *G*, RNA pull-down assay followed by biotin-labeled miR-27b-3p (wt or mutant) to examine whether miR-27b-3p is endogenously associated with MET. *H*, RIP assay was performed in OCI-LY3 cells, followed by RT-qPCR to determine bound miR-27b-3p associated with MET. ∗*p* < 0.05; ∗∗*p* < 0.01.

Based on the above results, lncRNA CHROMR might target miR-27b-3p to suppress RISC-mediated downstream mRNA silencing, we then examined the direct downstream target gene of miR-27b-3p. The target genes of miR-27b-3p were predicted by miRDB, miRTarBase, and TargetScan (Fig. 9*A*) (24, 25, 26), and the targeted gene function and pathways were enriched through Metascape (Fig. 9*B*) (27). Among the 14 genes, MET expression can be rescued by miR-27b-3p suppression in DLBCL (28, 29). TCGA data revealed that MET was highly expressed in 47 DLBCL tissues compared with 337 corresponding para-tumor specimens (Fig. 9C). MiR-27b-3p mimics (Fig. 9, *D* and *E*) significantly reduced mRNA and protein levels of MET, whereas miR-27b-3p inhibitor (Fig. 9, *D* and *E*) enhanced MET levels in DLBCL cells. RNA pull-down assay revealed the significant enrichment of MET using biotin-labeled miR-27b-3p (Fig. 9, *F* and *G*). RIP assay revealed that miR-27b-3p was highly enriched in the anti-MET group than in the IgG group (Fig. 9*H*). Therefore, MET might be a direct target of miR-27b-3p.

**Figure 10:**
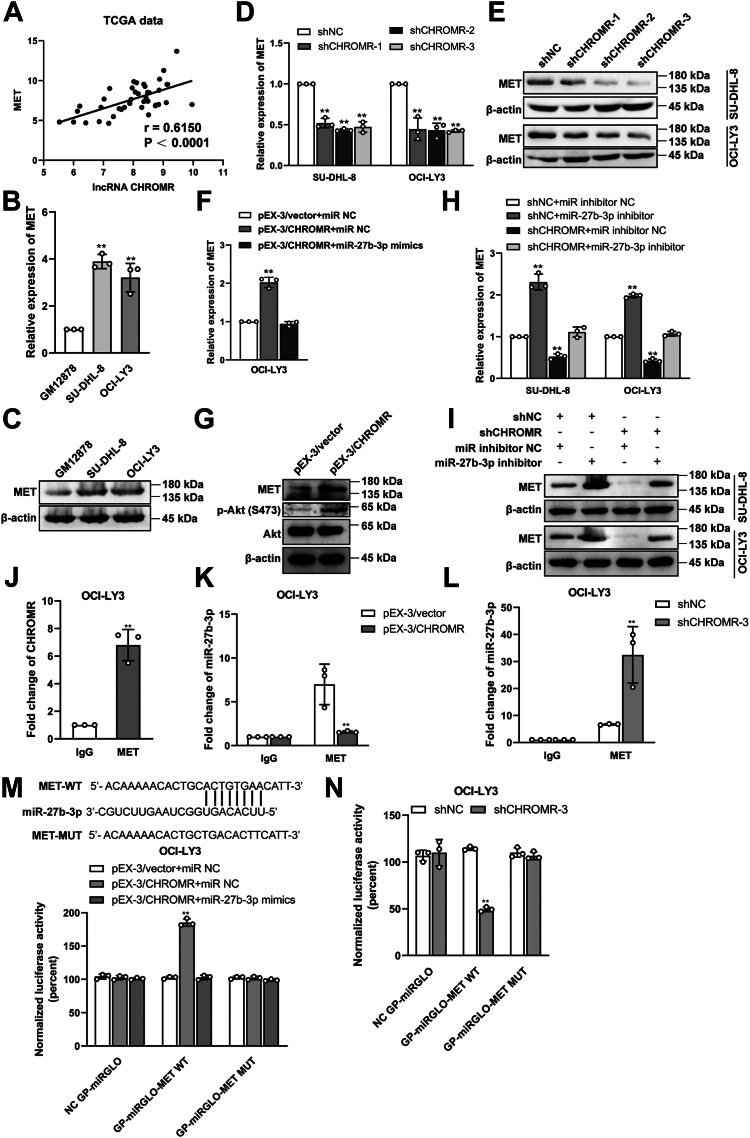
**LncRNA CHROMR targets MET by sponging with miR-27b-3p.***A*, correlation analysis between the mRNA levels of lncRNA CHROMR and MET in DLBCL tissues based on TCGA data. The expression of MET was analyzed by (*B*) RT-qPCR and (*C*) western blotting. *D* and *E*, effect of lncRNA CHROMR suppression on MET levels in DLBCL cells. *F*, expression levels of MET after indicated transfection. *G*, the protein levels of MET and AKT, and the phosphorylation level of AKT in indicated DLBCL cells were examined by western blotting. Expression levels of MET after indicated transfection by RT-qPCR (*H*) and western blotting (*I*). RIP assay was performed in (*J*) OCI-LY3 cells, (*K*) lncRNA CHROMR-overexpressed OCI-LY3 cells, (*L*) lncRNA CHROMR-depleted OCI-LY3 cells, followed by RT-qPCR to determine bound lncRNA CHROMR and miR-27b-3p associated with MET. *M* and *N*, Luciferase activity in DLBCL cells following the indicated transfection. Data were demonstrated as mean ± standard deviation of three independent experiments. ∗*p* < 0.05; ∗∗*p* < 0.01.

Based on these results, we hypothesized that a correlation might exist between lncRNA CHROMR and MET. Based on the RPISeq database, the interaction probabilities between lncRNA CHROMR and MET were listed as follows: RF = 0.75, SVM = 0.98, suggesting lncRNA CHROMR transcript had a high affinity with MET since RF > 0.5 and 0.57 < SVM < 0.99. MET expression positively correlated with lncRNA CHROMR levels (*p* < 0.0001, Fig. 10*A*). MET was highly expressed in SU-DHL-8 and OCI-LY3 cells compared with human B lymphocytes by RT-qPCR (Fig. 10*B*) and western blotting (Fig. 10*C*). Therefore, we further examined the influence of lncRNA CHROMR on MET. LncRNA CHROMR suppression (Fig. 10, *D* and *E*) significantly reduced mRNA and protein levels of MET, whereas CHROMR overexpression (Fig. 10, *F* and *G*) enhanced MET levels in DLBCL cells. The increase in MET expression by CHROMR overexpression was abolished by miR-27b-3p mimics (Fig. 10*F*). MET activates PI3K-AKT signaling (30). The phosphorylation level of AKT was elevated in lncRNA CHROMR-overexpressed OCI-LY3 cells (Fig. 10*G*) and reduced in lncRNA CHROMR-depleted DLBCL cells (Fig. 4*F*). Moreover, the decrease in MET expression by lncRNA CHROMR suppression was rescued by miR-27b-3p inhibitor, suggesting that lncRNA CHROMR targets MET level through sponging with miR-27b-3p (Fig. 10, *H* and *I*). RIP assay revealed that lncRNA CHROMR was highly enriched in the anti-MET group than in the IgG group (Fig. 10*J*). Then we wondered whether lncRNA CHROMR regulated the binding between MET and miR-27b-3p. MET bound less miR-27b-3p in lncRNA CHROMR-overexpressed OCI-LY3 cells than control cells (Fig. 10*K*), and bound more miR-27b-3p in lncRNA CHROMR-depleted OCI-LY3 cells than control cells (Fig. 10*L*). Dual luciferase reporter assay demonstrated that miR-27b-3p reduced the luciferase activity of MET-WT (wild type) in lncRNA CHROMR-overexpressed OCI-LY3 cells, but not the MET-MUT (mutant) (Fig. 10*M*). The luciferase activity of MET-WT was reduced in lncRNA CHROMR-depleted OCI-LY3 cells compared with that in the control cells (Fig. 10*N*). These data demonstrated that lncRNA CHROMR inhibited the binding between MET and miR-27b-3p.

### c-MET inhibitor PHA-665752 sensitized DLBCL cells to rituximab treatment

**Figure 11:**
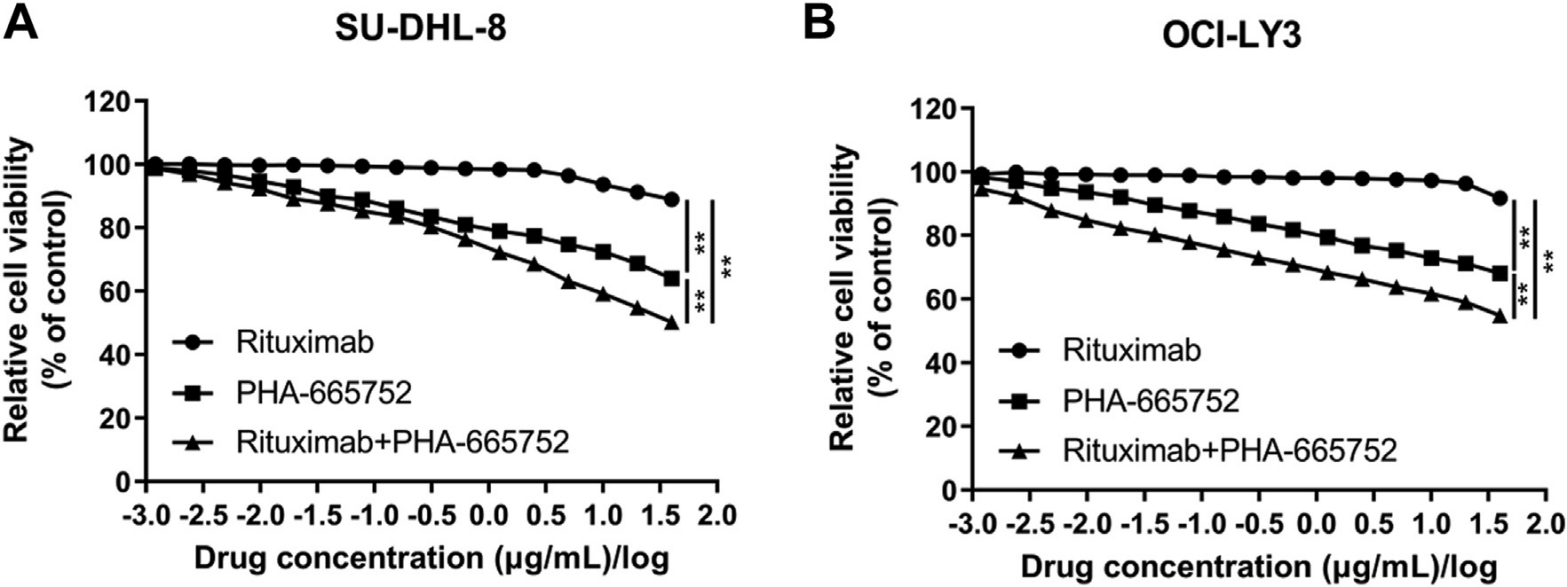
**c-MET inhibitor PHA-665752 sensitized DLBCL cells to rituximab treatment.** CCK-8 assay suggested synergism with PHA-665752 and rituximab combination in SU-DHL-8 cells (*A*) and OCI-LY3 cells (*B*). Data were demonstrated as mean ± standard deviation of three independent experiments. ∗*p* < 0.05; ∗∗*p* < 0.01.

To test the effects of MET inhibitors on rituximab response, we treated DLBCL cells with rituximab alone, c-MET inhibitor PHA-665752 alone or rituximab in combination with PHA-665752 for 72 h. The two-drug combination was more potent than the single-drug treatment in SU-DHL-8 and OCI-LY3 cells (Fig. 11). Collectively, c-MET inhibitor PHA-665752 sensitized DLBCL cells to rituximab treatment.

### LncRNA CHROMR promotes proliferation, invasion, and chemoresistance and represses apoptosis of DLBCL cells

**Figure 12:**
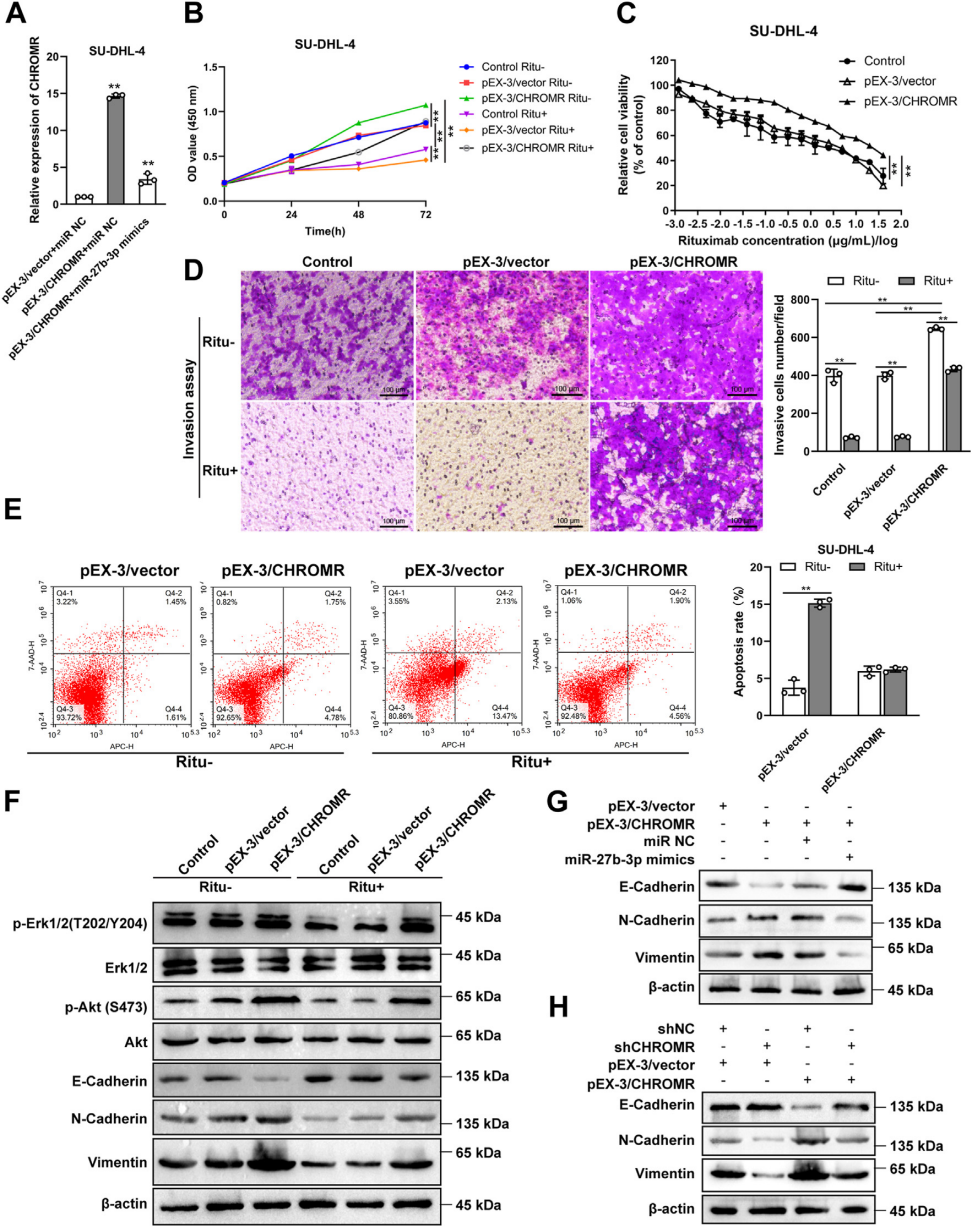
**The roles of lncRNA CHROMR and miR-27b-3p in tumorigenesis of DLBCL *in vitro*.***A*, the lncRNA CHROMR mRNA levels were determined by RT-qPCR. The cell proliferative or invasive ability was determined by (*B* and *C*) CCK8 assay or (*D*) transwell matrigel assay. Scale bar = 100 μm. *E*, cell apoptosis of DLBCL cells was measured by Annexin V-APC and 7-AAD staining, followed by flow cytometry. *F*, the expression of EMT marker proteins in lncRNA CHROMR-overexpressed and control SU-DHL-4 cells were detected by western blotting. *G* and *H*, the expression of EMT marker proteins after indicated transfection. Data were demonstrated as mean ± standard deviation of three independent experiments. ∗*p* < 0.05; ∗∗*p* < 0.01.

SU-DHL-4 cells were transfected with indicated plasmid and highly expressed lncRNA CHROMR was verified by RT-qPCR, and lncRNA CHROMR expression was reduced after co-transfected with lncRNA CHROMR inserts and miR-27b-3p mimics (Fig. 12*A*). LncRNA CHROMR overexpression enhanced rituximab resistance (5 μg/ml, Fig. 12*B*), promoted cell proliferative (Fig. 12*C*), and invasive abilities (Fig. 12*D*). SU-DHL-4 cells overexpressing lncRNA CHROMR were insensitive to rituximab treatment (Fig. 12*E*). In line with this, the phosphorylation levels of Erk1/2 and Akt were enhanced upon upregulated lncRNA CHROMR expression and stayed almost unchanged after rituximab treatment (20 μg/ml) (Fig. 12*F*). The downregulation of E-cadherin and upregulation of N-cadherin and vimentin stayed almost unchanged after rituximab treatment (20 μg/ml) upon highly expressed lncRNA CHROMR. Furthermore, the expression levels of N-cadherin, vimentin, and E-cadherin were reversed when plasmid pEX-3/CHROMR and miR-27b-3p mimics were co-transfected (Fig. 12*G*) or after transfected with plasmid pEX-3/CHROMR in SU-DHL-4 transfected with shCHROMR (Fig. 12*H*). Collectively, our data demonstrated that lncRNA CHROMR overexpression promotes proliferation, invasion, and inhibits apoptosis of DLBCL cells, whereas miR-27b-3p mimics significantly rescue these phenotypes.

## Discussion

LncRNA CHROMR is localized in chromosome 2q31.2 with five exons. LncRNA CHROMR has three transcript variants, and the transcript variant 1 has 2364 nucleotides in length. Although lncRNA CHROMR has been reported to regulate cholesterol efflux and HDL particle formation in primates (17, 18), the biological function and molecular mechanism of lncRNA CHROMR in DLBCL is largely unknown. Our data revealed that lncRNA CHROMR was highly expressed in rituximab-resistant cells and DLBCL tissues. Here, we revealed its oncogenic role in DLBCL tumorigenesis and chemoresistance. Using gain-of- function and loss-of-function assays, lncRNA CHROMR suppression inhibited DLBCL cell proliferation, invasion, enhanced DLBCL cell apoptosis, and sensitized DLBCL cells to rituximab treatment *in vitro*. Meanwhile, lncRNA CHROMR overexpression enhanced DLBCL cell proliferation, invasion, inhibited DLBCL cell apoptosis, and insensitized DLBCL cells to rituximab treatment *in vitro*. LncRNA CHROMR is distributed in both the nucleus and cytoplasm of DLBCL cells and promotes DLBCL progression through sponging with miR-27b-3p to regulate MET. We also examined whether lncRNA CHROMR suppression limits DLBCL tumor growth *in vivo*. SU-DHL-8 (1 × 10^7^ cells/mouse) or OCI-LY3 cells (1 × 10^7^ cells/mouse) with silenced lncRNA CHROMR or the negative control (shNC) were subcutaneously injected into the flanks of BALB/c (GemPharmatech) or NOD-SCID female mice (GemPharmatech). However, no visible tumors can be observed in NOD-SCID female mice. In BALB/c female mice, tumors in the shNC group did not grow further when reached 4 mm^3^ and tumors in the shCHROMR group gradually disappeared the third week after injection. In the future, we plan to try other cell lines to examine the functions of lncRNA CHROMR *in vivo*. The above experiments demonstrated that lncRNA CHROMR suppression reduced cell growth, invasion, and chemoresistance*.* LncRNA CHROMR acted as an oncogenic lncRNA in DLBCL.

Although rituximab has been reported to be effective in survival prolongation for DLBCL patients, acquired or inherent chemoresistance compromises its efficacy (7). LncRNA CHROMR suppression sensitized DLBCL cells to rituximab treatment. Furthermore, flow cytometry assay demonstrated that lncRNA CHROMR suppression enhanced rituximab-induced DLBCL cell apoptosis. Rituximab resistance could be due to changes in CD20 cell surface expression or conformational changes (31). LncRNA CHROMR overexpression reduced CD20 transcription in DLBCL cells. Furthermore, lncRNA CHROMR might regulate CD20 expression by phosphorylating HDAC3 in DLBCL cells. Collectively, lncRNA CHROMR suppression markedly reduced the phosphorylation levels of HDAC3, enhanced CD20 expression and sensitized DLBCL cells to rituximab, which suggests a promising therapeutic strategy for DLBCL patients with low CD20 expression. However, the clinical effectiveness and safety await further exploration.

For cytoplasmic lncRNAs, they act as miRNA sponges or competitive endogenous RNAs transcriptionally to regulate miRNA-mediated mRNAs targeting (32). For nuclear lncRNAs, they bind transcripts and promote alternative splicing of mRNAs (33). Some cytoplasmic and nuclear lncRNAs bind to and modulate the stability of cytoplasmic and nuclear proteins (32). Here, we revealed that lncRNA CHROMR was distributed in both the nucleus and cytoplasm of DLBCL cells and enhanced DLBCL progression by sponging miR-27b-3p.

Previous studies have suggested abnormal expression and regulation of miRNAs in many tumors (34, 35, 36, 37). MiRNAs are also involved in the proliferation, apoptosis, migration, invasion and chemoresistance in DLBCL (38, 39). Here, it was suggested that miR-27b-3p may be a direct target of lncRNA CHROMR. There were conflicting reports on if miR-27b acted as an oncogene (35, 40) or tumor suppressor (41, 42) in the development of human cancers in the literature. However, miR-27b was considered a tumor suppressor in DLBCL and was downregulated by Rel A/p65 (28). The qPCR results suggested the reciprocal inhibitory effect of lncRNA CHROMR and miR-27b-3p. The dual luciferase reporter assay revealed that miR-27b-3p was a direct target of lncRNA CHROMR. The RIP assay demonstrated that lncRNA CHROMR and miR-27b-3p are in the same RISC. LncRNA CHROMR inhibited the binding of miR-27b-3p to AGO2 and lncRNA CHROMR acts as a sponge for miR-27b-3p in DLBCL *via* the ceRNA mechanism. Furthermore, the effects of lncRNA CHROMR suppression on DLBCL cell proliferative and invasive abilities were rescued by miR-27b-3p inhibitor. There is no significant correlation between the expression of miR-27b-3p and survival of DLBCL patients according to ENCORI database (43) (Fig. S5). Collectively, these results demonstrated that lncRNA CHROMR sponges with miR-27b-3p in DLBCL.

In this study, MET was revealed as a direct target of miR-27b-3p, which is consistent with the previous finding that MET is a direct target of miR-27b-3p in DLBCL and MET expression can be restored by miR-27b-3p silencing (28, 29). The expression levels of both lncRNA CHROMR and MET were upregulated in most DLBCL cell lines, which were consistent with online prediction. A positive correlation between lncRNA CHROMR and MET expression was verified in DLBCL tissues from the TCGA database. LncRNA CHROMR promoted MET abundance in DLBCL cells, while miR-27b-3p demonstrated an opposite regulatory effect. MET downregulation by lncRNA CHROMR suppression was dependent on the upregulation of miR-27b-3p *via* the cytoplasmic ceRNA mechanism. LncRNA CHROMR was demonstrated to interact with MET and stabilize the expression level of MET in DLBCL. RIP assay and RNA pull-down assay revealed that MET interacted with miR-27b-3p in DLBCL cells and lncRNA CHROMR inhibited the binding between MET and miR-27b-3p. The luciferase activity of MET-WT was reduced in lncRNA CHROMR-depleted DLBCL cells and enhanced in lncRNA CHROMR-overexpressed DLBCL cells, and the increase was abolished by miR-27b-3p mimics transfection by dual luciferase reporter assay, suggesting that lncRNA CHROMR stabilizes the expression of MET by sponging with miR-27b-3p.

In this study, we found that MET activates AKT signaling and the phosphorylation level of AKT was elevated upon lncRNA CHROMR overexpression and reduced upon lncRNA CHROMR suppression. Previous studies demonstrated that PI3K/Akt dysregulation was observed in 55% of the GCB-DLBCLs and 14% of the non-GCB DLBCLs with PTEN loss (44). Rituximab resistance in DLBCL was correlated with constitutively activated apoptotic pathways, including the PI3K/AKT signaling pathway (45, 46). PI3K/AKT suppression reverses R-CHOP resistance in DLBCL (47). Highly expressed p-AKT predicted worse progression-free survival (PFS) and overall survival (OS) (45). Another potential mechanism of R-CHOP resistance in DLBCL, cell adhesion-mediated drug resistance (CAM-DR), is also correlated with dysregulated PI3K-Akt signaling (48, 49). Moreover, c-MET inhibitor PHA-665752 sensitized DLBCL cells to rituximab treatment. Collectively, increased MET and AKT was correlated with rituximab resistance of DLBCL, which was consistent with our results that the phosphorylation level of AKT was elevated upon lncRNA CHROMR overexpression.

In the future, we would analyze the levels of lncRNA CHROMR in DLBCL tumors from patients who are not resistant to rituximab after collecting enough specimens to further support the idea that this overexpression of lncRNA is associated with rituximab resistance. Meanwhile, we would find other more appropriate DLBCL cell lines with constitutive overexpression of lncRNA CHROMR and not the ones with decreased expression to support the concept that lncRNA CHROMR truly promotes DLBCL development *in vivo* straightforwardly, and not only through *in vitro* experiments.

**Figure 13:**
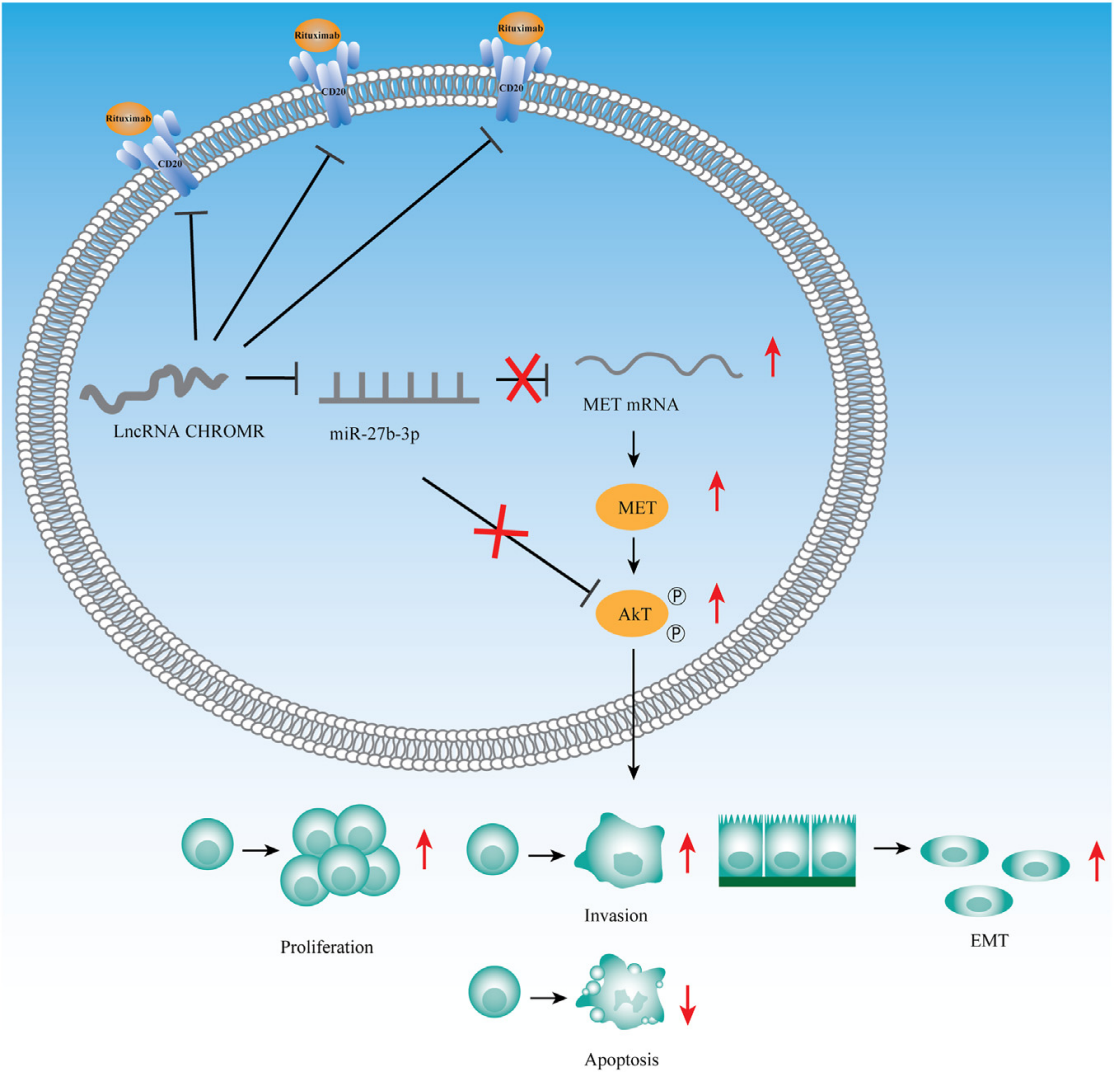
**Schematic representation of the molecular mechanism of “lncRNA CHROMR/miR-27b-3p/MET” axis in DLBCL**.

To conclude, the study revealed lncRNA CHROMR suppression enhances miR-27b-3p inhibition on MET level *via* the ceRNA mechanism (Fig. 13). Our data contribute to a deepened understanding of lncRNA CHROMR in DLBCL progression. LncRNA CHROMR might serve as a promising diagnostic and therapeutic target for DLBCL.

## Experimental procedures

### Cell lines, plasmids and inhibitors

Human B lymphocyte GM12878 was purchased from HyCyte. Human DLBCL cell lines OCI-LY3, OCI-LY19 and SU-DHL-4 were purchased from Beina Chuanglian Biological Research Institute. Human DLBCL cell lines RC and SU-DHL-8 were purchased from the American Type Culture Collection (ATCC). All these cell lines were cultured in RPMI-1640 medium (Gibco, Thermo Fisher Scientific) with 10% FBS (Gibco). All these cell lines were authenticated using short tandem repeat (STR) matching analysis. The lentiviral vectors of lncRNA CHROMR (short hairpin RNA (shRNA)- CHROMR), the miR-27b-3p inhibitor and mimics were designed and purchased from GenePharma. The sequence information is shown in Table 1. The lentiviral vector plasmid used was LV3 (H1/GFP&Puro). The plasmid DNA (pEX-3/CHROMR) with the restriction enzyme cutting site XhoI/NotI was obtained from GenePharma. The GP-miRGLO-CHROMR-WT (wild-type) and its corresponding mutants, and the GP-miRGLO-MET-WT (wild-type) and its corresponding mutants were synthesized and purchased from GenePharma. HDAC3 inhibitor RGFP966 and MET inhibitor PHA-665752 were purchased from Selleck Chemicals.

**Table 1:** Sequence information

Name	Sense sequence	Antisense sequence
miR-27b-3p mimics	UUCACAGUGGCUAAGUUCUGC	AGAACUUAGCCACUGUGAAUU
NC mimics	UUCUCCGAACGUGUCACGUTT	ACGUGACACGUUCGGAGAATT
miR-27b-3p inhibitor	GCAGAACUUAGCCACUGUGAA	
Inhibitor NC	CAGUACUUUUGUGUAGUACAA	

NC, negative control.

### RNA extraction and real-time PCR

Total RNA was extracted using TRIzol reagent (Takara). The cDNA was synthesized from lncRNA or miRNA using PrimeScript RT reagent Kit with gDNA Eraser (Takara) or Mir-X miRNA First-Strand Synthesis Kit (Takara). Then, qPCR was applied to calculate the relative expression levels of lncRNA CHROMR and miRNAs using TB Green Premix Ex Taq II (Takara) by the 2^-ΔΔCt^ method on StepOnePlus Real-Time PCR System (Applied Biosystems). The expression levels of lncRNA CHROMR and miRNA were normalized to *GAPDH* and small nucleolar RNA *U6*, respectively. The primers used are listed in Table 2.

**Table 2:** Primers and sequences

Primers	Sequences (5′-3′)
CHROMR-F	CCGCGGTGATTTAACAGCTT
CHROMR-R	AGCTCCTGCTCGTTCTTCAG
MET-F	CTCCCATCCAGTGTCTCCAGAA
MET-R	GGAAATGTCTGCAGCCCAAG
CD20-F	TTGAACGGTGAGGTGTCAGGTC
CD20-R	TTCTCAGGCCTGCTCCTACTATGTC
GAPDH-F	GCACCGTCAAGGCTGAGAAC
GAPDH-R	TGGTGAAGACGCCAGTGGA
hsa-miR-27b-3p-F	CAGTGGCTAAGTTCTGCAAA
hsa-miR-27b-3p-R	Universal Reverse Primer (Takara)
hsa-miR-128-3p-F	TCACAGTGAACCGGTCTCTTTAA
hsa-miR-128-3p-R	Universal Reverse Primer (Takara)
hsa-miR-33a-5p-F	GTGCATTGTAGTTGCATTGCAAA
hsa-miR-33a-5p-R	Universal Reverse Primer (Takara)
hsa-miR-33b-5p-F	CATTGCTGTTGCATTGCAAAA
hsa-miR-33b-5p-R	Universal Reverse Primer (Takara)
U6-F	GGAACGATACAGAGAAGATTAGC
U6-R	TGGAACGCTTCACGAATTTGCG

### Transfection

Stable shRNA-mediated lncRNA CHROMR suppression cell lines were selected using 6 μg/ml puromycin. The three shRNA sequences targeting CHROMR were as follows:

shRNA-CHROMR-1: 5′-TTGCTTCCTAAAGGGTCTTTA-3′;

shRNA-CHROMR-2: 5′-TTGAGGAACTGAGAAGCTTAT-3′;

shRNA-CHROMR-3: 5′-GAGCTGAGACTTCCACCTAAT-3′.

The LV3 NC sequence was: 5′-TTCTCCGAACGTGTCACGT-3′. (NC: negative control).

The miR-27b-3p inhibitor and mimics were transfected using siRNA-mate (GenePharma). Cells were harvested 48 h after transfection for subsequent experiments.

Plasmid transfection were performed according to the manufacturer’s protocol.

### Cell proliferation, apoptosis and transwell invasion assays

CCK-8 (Beijing Biosynthesis Biotechnology Co, Ltd) assay was conducted to examine cell viability. Annexin V-APC/7-AAD Apoptosis Detection Kit (KeyGEN BioTECH) was applied to examine the cell apoptosis. Transwell invasion assay was conducted to examine cell invasion. The transwell inserts (8 μm pore size; Corning) were coated with 40 μl of Matrigel (1:8 mixed with FBS-free medium; Corning). 400 μl of 2 × 10^5^ indicated DLBCL cells were plated in the upper chamber of inserts without serum. Medium with 20% FBS was added into the lower chamber. After culture for 48 h, the cells on the bottom chamber were fixed, stained, counted, and calculated. Cells with indicated treatment were seeded into 6-wells plates (1000 cells/well) and cultured for 5 days. The number of colonies (>50 cells) was counted.

### Flow cytometry analysis of CD20 staining

For analysis of cell CD20 staining, OCI-LY3 cells (1 × 10^7^ cells) were collected and incubated with rabbit IgG isotype control (AC042, 1:50, ABclonal) or CD20 rabbit mAb (A4893, 1:50, ABclonal) for 30 min at room temperature, followed by goat anti-rabbit pAb Alexa Fluor 647 (AS086, ABclonal) staining for 30 min at room temperature. Non-fluorescently stained OCI-LY3 cells were used as blank control. The expression levels of CD20 were detected with an Agilent NovoCyte flow cytometer (Agilent). Each experiment was conducted three times.

### Dual luciferase reporter assay

Luciferase vectors, miR-27b-3p mimics or miR-27b-3p NC were co-transfected into OCI-LY3 or HEK-293T cells using Lipofectamine 2000 (Invitrogen). The dual luciferase reporter gene assay kit (GenePharma) was performed according to the manufacturer’s protocol.

### Subcellular fractionation assay

Nuclear and cytoplasmic RNAs in DLBCL cells were isolated using the PARIS Kit (Life Technologies), reversely transcribed, and followed by RT-qPCR as described above.

### RNA immunoprecipitation (RIP) assay

RNA Immunoprecipitation Kit (Geneseed) was performed for the RIP assay. Cell extracts were incubated with magnetic beads conjugated with Ago2 antibody (#2897, Cell Signaling Technology), MET antibody (SC-514148, Santa Cruz Biotechnology) or IgG antibody (AC005, ABclonal) at 4 °C overnight. RNAs and proteins in the precipitates were detected using RT-qPCR and western blotting, respectively.

### RNA pull-down assay

OCI-LY3 cells were transfected with biotinylated miR-27b-3p mimics (Ribobio) or NC. After 48 h, cell lysates were incubated with streptavidin magnetic beads (MCE). LncRNA CHROMR mRNA levels or MET protein levels were detected by RT-qPCR or Western blot.

### Western blotting

Protein extraction, separation, and visualization were conducted as described previously (11, 12, 13). Membranes were incubated with primary antibodies against CD20 (1:1000; #48750), MET (1:1000; #8198), Erk1/2 (1:1000; #4695), p-Erk1/2 (1:1000; #4370), Akt (1:1000; #4691), p-Akt (1:1000; #4060), E-Cadherin (1:1000; #3195), N-Cadherin (1:1000; #13116), Vimentin (1:1000; #5741), HDAC3 (1:1000; #85057), p-HDAC3 (1:1000; #3815) from Cell Signaling Technology, and β-actin (1:2500; TA-09) from ZSGB-Biotechnology.

### Statistical analysis

Statistical analyses were conducted using the SPSS 19.0 statistical software (SPSS, Inc) and the GraphPad Prism v8.0 (GraphPad, Inc). Comparison between groups was analyzed using independent *t* test or one-way ANOVA and *p* < 0.05 was considered statistically significant. ∗*p* < 0.05; ∗∗*p* < 0.01.

## Data availability

The datasets generated during and/or analyzed during the current study are available from the corresponding author on reasonable request.

## Supporting information

This article contains supporting information.

## Conflict of interest

The authors declare that they have no conflict of interest.
