# Supplementary material for: LncRNA CHROMR/miR-27b-3p/MET axis promotes the proliferation, invasion, and contributes to rituximab resistance in diffuse large B-cell lymphoma
Source: J Biol Chem. 2024 Feb 16;300(3):105762. doi: 10.1016/j.jbc.2024.105762 (PMC10940993; doi:10.1016/j.jbc.2024.105762)
Supplement: Supplemental Figures S1–S5 [file mmc1.doc]

**LncRNA CHROMR/miR-27b-3p/MET axis promotes the proliferation, invasion and contributes to rituximab resistance in diffuse large B-cell lymphoma**

**Supporting Information**

**Supplementary Figures**


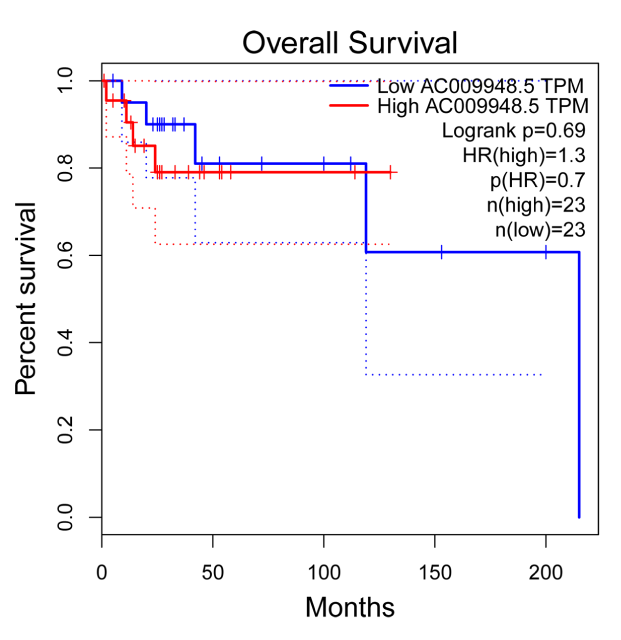


**Supplementary Figure 1.**

Survival analyses of the correlation between lncRNA CHROMR expression and overall survival of DLBCL patients. *n* = 46, *P* = 0.69 by log-rank test.


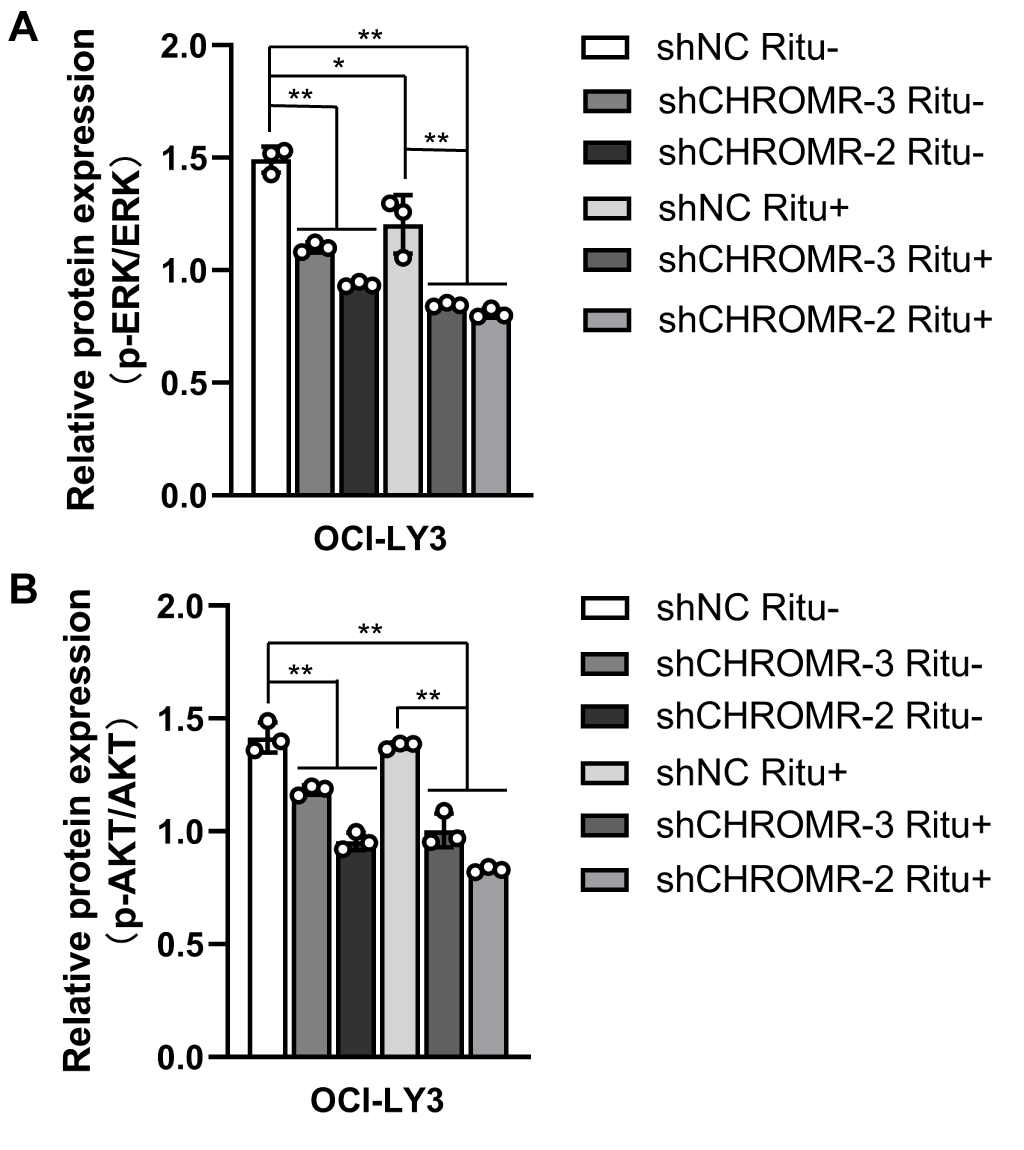


**Supplementary Figure 2.**

The relative phosphorylation levels of ERK **(A)** and AKT **(B)** in Figure 4F were calculated from three independent experiments. *, *P*＜0.05; **, *P*＜0.01.


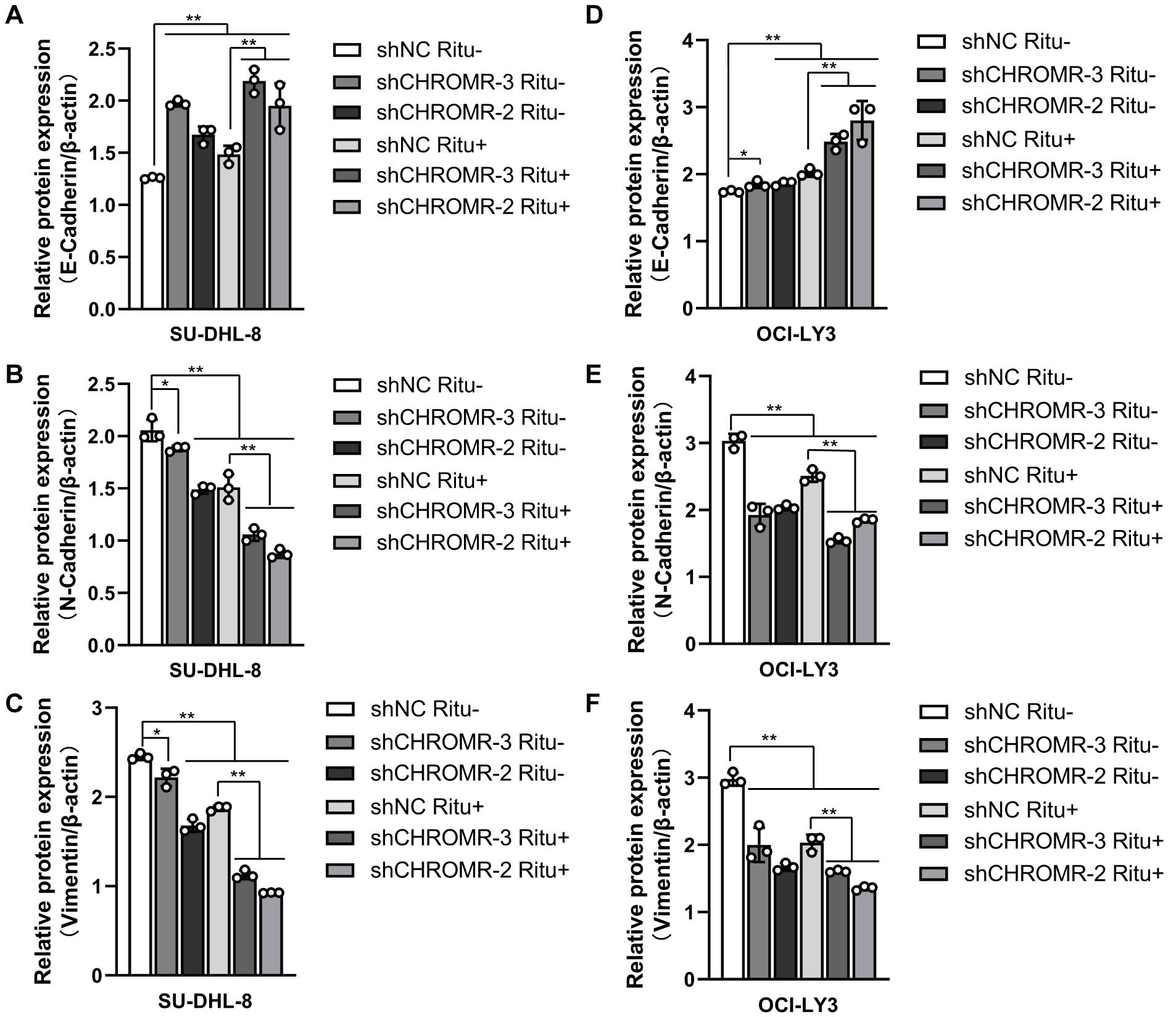


**Supplementary Figure 3.**

The relative expression levels of E-cadherin, N-cadherin and vimentin in Figure 5C were calculated from three independent experiments. **(A-C)** The relative expression levels of E-cadherin, N-cadherin and vimentin in SU-DHL-8 cells were calculated from three independent experiments. **(D-F)** The relative expression levels of E-cadherin, N-cadherin and vimentin in OCI-LY3 cells were calculated from three independent experiments. *, *P*＜0.05; **, *P*＜0.01.


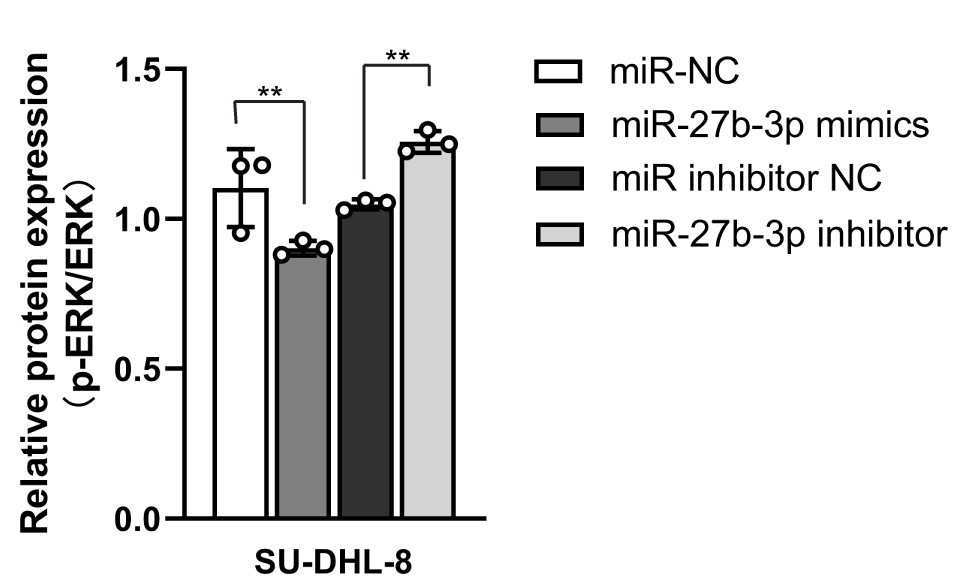


**Supplementary Figure 4.**

The relative phosphorylation levels of ERK in Figure 8A were calculated from three independent experiments in SU-DHL-8 cells. *, *P*＜0.05; **, *P*＜0.01.


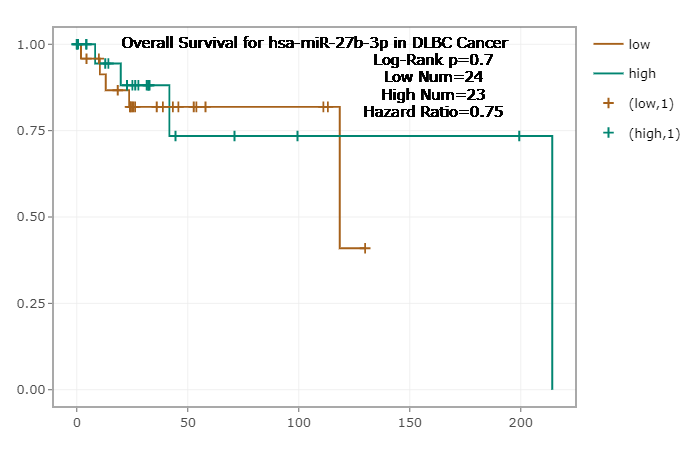


**Supplementary Figure 5.**

The survival analyses of the correlation between miR-27b-3p expression and overall survival of DLBCL patients.
